# Fabrication and Application of a 3D-Printed Poly-*ε*-Caprolactone Cage Scaffold for Bone Tissue Engineering

**DOI:** 10.1155/2020/2087475

**Published:** 2020-01-30

**Authors:** Siyi Wang, Rong Li, Yongxiang Xu, Dandan Xia, Yuan Zhu, Jungmin Yoon, Ranli Gu, Xuenan Liu, Wenyan Zhao, Xubin Zhao, Yunsong Liu, Yuchun Sun, Yongsheng Zhou

**Affiliations:** ^1^Department of Prosthodontics, Peking University School and Hospital of Stomatology, Beijing 100081, China; ^2^Center of Digital Dentistry, Peking University School and Hospital of Stomatology, Beijing 100081, China; ^3^National Engineering Laboratory for Digital and Material Technology of Stomatology, National Clinical Research Center for Oral Diseases, Beijing Key Laboratory of Digital Stomatology, Beijing 100081, China; ^4^Dental Medical Devices Testing Center, Peking University School and Hospital of Stomatology, Beijing 100081, China; ^5^Department of Materials Science and Engineering, College of Engineering, Peking University, Beijing 100871, China; ^6^Department of Prosthodontics, Yinchuan Stomatology Hospital, Yinchuan, Ningxia 750002, China

## Abstract

Poly-ε-caprolactone (PCL) is a promising synthetic material in bone tissue engineering (BTE). Particularly, the introduction of rapid prototyping (RP) represents the possibility of manufacturing PCL scaffolds with customized appearances and structures. Bio-Oss is a natural bone mineral matrix with significant osteogenic effects; however, it has limitations in being constructed and maintained into specific shapes and sites. In this study, we used RP and fabricated a hollow-structured cage-shaped PCL scaffold loaded with Bio-Oss to form a hybrid scaffold for BTE. Moreover, we adopted NaOH surface treatment to improve PCL hydrophilicity and enhance cell adhesion. The results showed that the NaOH-treated hybrid scaffold could enhance the osteogenesis of human bone marrow-derived mesenchymal stem cells (hBMMSCs) both *in vitro* and *in vivo*. Altogether, we reveal a novel hybrid scaffold that not only possesses osteoinductive function to promote bone formation but can also be fabricated into specific forms. This scaffold design may have great application potential in bone tissue engineering.

## 1. Introduction

Bone defects resulting from congenital deformity, trauma, inflammation, and tumor resection can cause patients great pain and impose a tremendous burden on health-care systems. Thus, bone substitutes are commonly used for bone regeneration; for example, Bio-Oss is a porous, nonantigenic, natural bone mineral matrix acquired by removing all organic components of the bovine bone, which exhibits a significant local osteogenic effect [1, 2]. However, it can successfully maintain the shape of the defect when implanted into small-scale bone defects, but struggles on larger defective areas.

Bone tissue engineering (BTE) is now providing a new option for the therapy of bone defects. The method involves combining porous biocompatible scaffolds with donor cells to promote bone regeneration, where human bone marrow-derived mesenchymal stem cells (hBMMSCs) are the most widely used [3]. Various conventional techniques are involved in the scaffold manufacturing process, such as phase separation, freeze-drying, solvent casting, and gas formation. However, these methods only manufacture randomly formed scaffolds, with poor control of the internal architecture [4]. The introduction of rapid prototyping (RP) technology represents the possibility of producing customized scaffolds with specific 3D geometry, internal structure, and architecture. Also, such scaffolds can be customized and adapted to complex bone defect areas [5, 6]. Fused deposition modeling (FDM) is a type of RP technology, based on extrusion of molten polymer materials. Compared with other RP techniques, FDM shows great priority in flexibility and material processing [7].

Poly-*ε*-caprolactone (PCL) is a Food and Drug Administration (FDA)-approved polymer, commonly used as the raw material of BTE scaffolds. It exhibits appropriate mechanical strength, low melting point (55–60°C), good compatibility, and biodegradable properties [8, 9].

In this study, using FDM, we fabricated hollow, porous, cage-shaped PCL scaffolds. The cages were surface-modified with NaOH and loaded with Bio-Oss to form a hybrid BTE scaffold for *in vivo* studies. We assessed whether surface treatment with NaOH influenced proliferation and osteogenic differentiation of hBMMSCs. We also investigated whether our NaOH-treated hybrid BTE scaffolds could be used to promote bone formation *in vivo*.

## 2. Materials and Methods

### 2.1. Scaffold Preparation

We obtained PCL (number average molecular weight; Mn = 37,000 Da) from Shinuo Technology Co., Ltd (China). Following the FDM technique, PCL scaffolds were computer-aided designed (CAD) using Geomagic Studio 2012 software (Raindrop, USA) and fabricated using the Elements Mixture-I bioprinter and its supporting slicing software (Shinuo Technology Co., Ltd., China). We used a nozzle size of 300 *μ*m and a feed rate of 500 mm/min. We prepared PCL sheet scaffolds of 33 mm diameter × 2 mm thickness; 21 mm diameter × 2 mm thickness; and 14 mm diameter × 2 mm thickness for our *in vitro* cell studies. We set the filament distance (FD) at 300 *μ*m. Layers were 100, 200, and 300 *μ*m thick, and the lay-down pattern was 0/90°. We next imported mandible STL data into the Geomagic Studio 2012 software, cut a section, and acquired PCL cage scaffolds as hollow structures using the bioprinter. The cage scaffolds measured 12 × 12 × 6 mm^3^ in size, with a 0/90° lay-down pattern and an FD of 300 *μ*m. The top floor of the cage could be cut open as a lid, and in the subsequent experiments, Bio-Oss was loaded into these cage scaffolds and then the lids were closed.

### 2.2. Morphological Characterization

We investigated the surface morphology of scaffolds with different layer thicknesses after they were installed onto aluminum stubs and coated with gold. We observed the samples using scanning electron microscopy (SEM; S-4800, Hitachi, Japan).

### 2.3. hBMMSC Culture and Osteogenic Induction

We purchased hBMMSCs from ScienCell (San Diego, CA, USA). We grew cells in proliferation medium (PM), comprising fresh a-minimum essential medium (a-MEM), 10% (v/v) fetal bovine serum (FBS), 100 U/mL penicillin G, and 100 mg/mL streptomycin. Then, we added 10 mM *β*-glycerophosphate, 10 nM dexamethasone, and 50 *μ*g/ml L-ascorbic acid to the PM to constitute osteogenic medium (OM). We used an incubator with a controlled environment (95% air, 5% CO_2_, 37°C, and 100% relative humidity) for cell culture. We conducted all subsequent experiments in triplicate.

### 2.4. In Vitro Cytotoxicity of PCL Scaffolds

We assessed PCL cytotoxicity according to the ISO10993-5 standard protocol [10]. Briefly, we fabricated PCL films using the FDM technique and incubated them in PM for 24 h at 37°C. We used an extraction ratio of 6 cm^2^/mL, before filtering the supernatant using a 0.20-*μ*m filter for sterilization. We cultured cells using PM in a 24-well plate (1 × 10^4^/well) for 24 h. We then replaced PM using 500 *μ*L fluid extractions. After incubating for 2 h, we quantified cell proliferation activity using the Cell Counting Kit-8 assay (CCK-8; Dojindo Laboratories, Kumamoto, Japan). We observed absorbance readings using a microplate reader (ELx 800, BioTek, USA) at 450 nm at 0, 1, 3, 5, and 7 days.

To evaluate *in vitro* cytotoxicity of PCL scaffolds, we used the Live/Dead assay and a confocal Zeiss Axiovert 650 microscope (Carl Zeiss MicroImaging, Oberkochen, Germany). We used 14 mm diameter × 2 mm thick scaffolds in our experiment. We sterilized the scaffolds for 1 h with 75% ethanol and immersed them in PM at 37°C for 24 h, before placing them into a 24-well plate. We seeded 500 *μ*L cell suspension onto each scaffold sheet at a density of 1 × 10^6^ cells/mL. After incubation at 37°C for 12 h, we immersed the scaffolds in PBS with 4 mM calcein acetoxymethyl ester (calcein AM) and 16 mM propidium iodide (PI) for 30 min. We detected PI (dead cells = red) and calcein AM (live cells = green) fluorescence at excitation wavelengths of 568 and 488 nm, respectively.

### 2.5. Surface Modification Evaluated by SEM and Contact Angle

We conducted NaOH treatment by soaking the scaffold samples in 3M NaOH for 24 h at room temperature. We then characterized the surface morphology of both untreated and treated scaffolds using SEM. We evaluated hydrophilicity using the contact angle system (Kino Industry, New York, USA). Briefly, we added a water droplet (1 *μ*L) on the scaffold surface and measured the water contact angle after 30 s.

### 2.6. Mechanical Properties

We measured compressive mechanical properties using a universal testing machine (Instron 5969, Instron, USA) according to the American Society for Testing and Materials (ASTM) standard D695-02a protocol. Both treated and untreated groups contained 5 cylindrical samples (5 mm diameter × 10 mm height). In the compression tests, the loading rate was 1 mm/min until the sample reached 70% of the original height. In the fitted stress-strain curve, we calculated the slope of the initial linear region as the elastic modulus [11].

### 2.7. Cell Adhesion and Proliferation Assay

We seeded 500 *μ*L cell suspension onto scaffold sheets of 14 mm diameter × 2 mm thickness in a 24-well plate at a density of 1 × 10^6^ cells/mL. After incubation for 12 h, we washed the scaffolds twice with PBS and fixed them in 4% paraformaldehyde for 10 min, before incubating with fluorescein isothiocyanate (FITC)-labeled phalloidin for 30 min. We then viewed the scaffolds using a confocal microscope at 488 nm wavelength.

To study the effects of treated and untreated PCL scaffolds on cell proliferation and viability, we seeded hBMMSCs onto the sheets in a 24-well plate and conducted CCK-8 assay after culturing for 0, 1, 3, 5, and 7 days. We used a microplate reader to measure the OD (absorbance) value of each well at 450 nm.

### 2.8. Alkaline Phosphatase (ALP) Activity of hBMMSCs

We seeded cells at a density of 2 × 10^6^ cells/mL on scaffold sheets of 21 mm diameter × 2 mm thickness, both treated and untreated, in 12-well plates. We divided them into four groups as follows: (1) untreated scaffolds cultured in PM (PCL + PM), (2) treated scaffolds cultured in PM (PCL-NaOH + PM), (3) untreated scaffolds cultured in OM (PCL + OM), and (4) treated scaffolds cultured in OM (PCL-NaOH + OM). We conducted ALP staining 7 days after osteoinduction (OI) using the nitroblue tetrazolium (NBT)/5-bromo-4-chloro-3-indolyl phosphate (BCIP) staining kit (CoWin Biotech, China).

We quantified ALP activity at the same time point. We seeded cells on 33 mm diameter × 2 mm thick sheets in 6-well plates at a density of 5 × 10^6^ cells/mL. 7 days after OI, we added 1 mL 1% Triton X-100 for 30 min at 4°C to lyse the cells, before measuring the total protein content using the BCA protein assay kit (Thermo Fisher Scientific). We measured ALP activity using the ALP activity assay kit (Nanjing Jiancheng Bioengineering Institute). We measured absorbance using a microplate reader at 520 nm wavelength.

### 2.9. Alizarin Red S (AR-S) Staining and Quantification of Mineralization

We seeded cells on sheets in 12-well plates, which were divided into the four aforementioned groups. 14 days after OI, we fixed the samples for 30 min, before staining with 1% AR-S in dH_2_O (pH 4.2) for 20 min. After staining, we washed the samples three times with dH_2_O, before incubating in 100 mM cetylpyridinium chloride for 1 h to release any calcium-bound alizarin red S into the solution. We quantified matrix mineralization by measuring absorbance at 562 nm wavelength.

### 2.10. Quantitative Real-Time PCR

We seeded cells on sheets in 6-well plates, divided into the aforementioned four groups. 7 and 14 days after OI, we extracted total RNA from hBMMSCs using the Trizol reagent (Invitrogen, Carlsbad, CA, USA). We determined total RNA concentration using a NanoDrop 8000 spectrophotometer (Pierce Thermo Scientific). We used the PrimeScript RT reagent kit (Takara, Tokyo, Japan) to conduct reverse transcription. We also used the Power SYBR Green PCR Master Mix and the ABI PRISM 7500 sequence detection system (Applied Biosystems, Foster City, CA) to quantify gene expression. We used GAPDH as the reference gene. We used the primers listed in Table 1.

### 2.11. Immunofluorescent Staining for OCN

We seeded cells on scaffold sheets in 24-well plates, divided into two groups: (1) untreated scaffolds cultured in OM (PCL + OM) and (2) scaffolds treated with 3 M NaOH cultured in OM (PCL-NaOH + OM). 14 days after OI, we incubated the samples overnight at 4°C in 1 : 200 anti-osteocalcin primary antibodies (Santa Cruz, Dallas, TX, USA) and then incubated them in 1 : 500 specified secondary antibody (Cell Signaling Technology, Beverly, MA, USA) for 1 h. We finally viewed the samples using a confocal microscope at 488 nm wavelength.

### 2.12. Ectopic Bone Formation In Vivo

This study was approved by the Institutional Animal Care and Use Committee of the Peking University Health Science Center (LA2014233), and all animal experiments were performed in accordance with the institutional animal guidelines. The BALB/c nude mice were purchased from Vital River Corporation (Beijing, China) and allowed free access to water and a maintenance diet, with room temperature at 21 ± 2°C and in a 12 hour light/dark cycle.

We applied hollow PCL cage scaffolds in this assay. We divided the scaffolds into two groups (*n* = 6 for each group): (1) PCL cage scaffolds loaded with Bio-Oss and hBMMSCs (PCL-Bio-Oss + cells) and (2) PCL cage scaffolds treated with 3 M NaOH loaded with Bio-Oss and hBMMSCs (PCL-NaOH-Bio-Oss + cell). We seeded hBMMSCs onto the scaffolds at a density of 5 × 10^6^ cells/mL. For general anesthesia, pentobarbital sodium (50 mg/kg) was given to the mice by intraperitoneal injections. We implanted all scaffolds into the dorsal subcutaneous space of 6-week-old female BALB/c nude mice. We sacrificed the mice 8 weeks after implantation, harvested the specimen, and fixed them for one week.

### 2.13. Histological Analysis

We decalcified the samples in 10% EDTA (pH 7.4) for 2 weeks, before dehydrating them and embedding them in paraffin. We cut 5 *μ*m cross sections from the middle of the scaffolds and performed hematoxylin and eosin (H&E) and Masson's trichrome staining. We then conducted immunohistochemical (IHC) analysis of OCN expression to evaluate osteogenesis.

### 2.14. Statistical Analysis

We analyzed data using SPSS Statistics 20.0 software (IBM) applying one-way analysis of variance. Results are presented as means ± standard deviations. We considered *p* < 0.05 as statistically significant.

## 3. Results

### 3.1. Morphologies and Cytotoxicity of PCL Scaffolds

**Figure 1:**
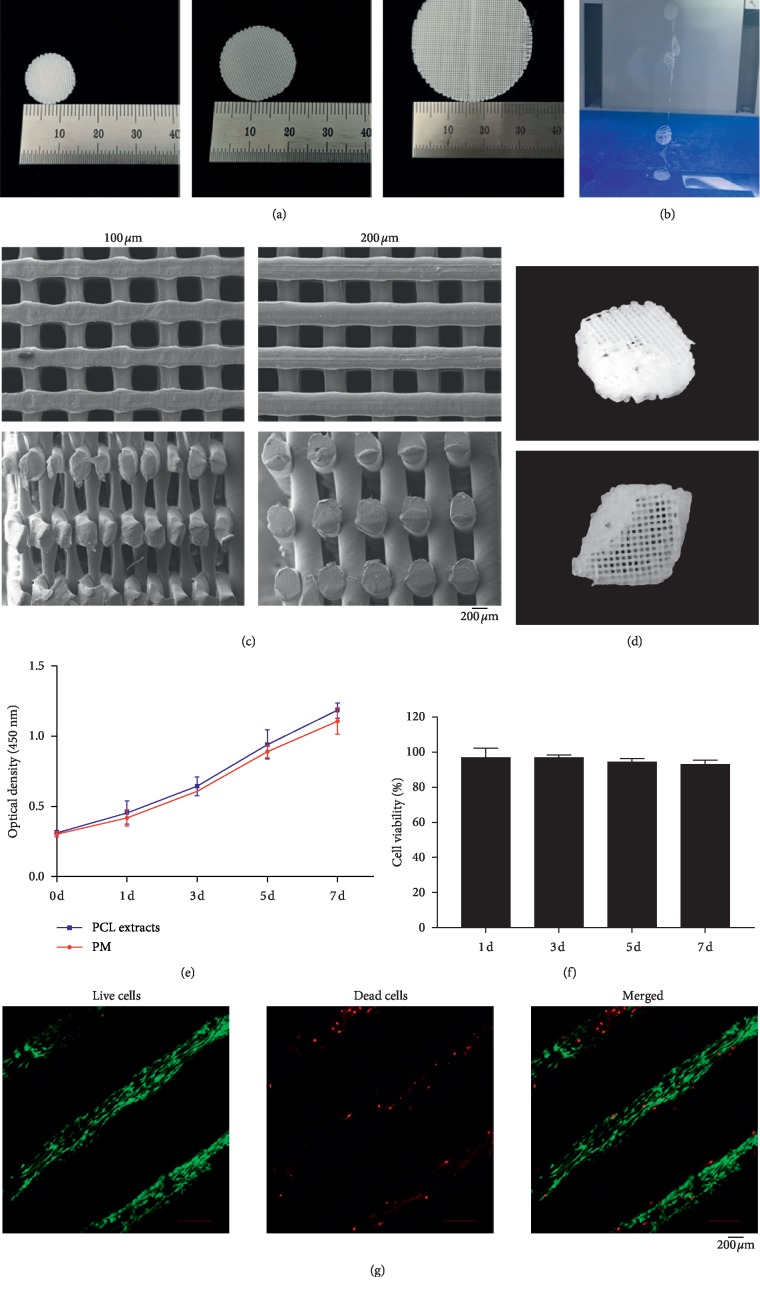
Morphologies and cytotoxicity of PCL scaffolds. (a) Fabrication of PCL sheets with diameters of 14 mm, 21 mm, and 33 mm. (b) PCL sheets with 300 *μ*m-thick layers could not attach tightly. (c) Surface and cross-sectional morphologies of PCL sheets with 100 *μ*m and 200 *μ*m-thick layers. (d) Fabrication of PCL cage scaffolds using the FDM technique, with 200 *μ*m-thick layers. (e) Proliferation curves of hBMMSCs cultured with PM and PCL extracts. Mean ± SD; *n* = 3. (f) Cell viability of hBMMSCs cultured with extraction fluid of PCL compared with PM. Mean ± SD; *n* = 3. (g) Live/dead staining of hBMMSCs cultured on treated and untreated PCL sheets for 12 h. Nuclei and mitochondria are colored green, and damaged cells are colored red. Bar represents 200 *μ*m for (c) and (f).

We analyzed fiber morphologies of prepared PCL sheets with 100, 200, and 300 *μ*m-thick layers. In the 300 *μ*m group (Figure 1(a)), we noted that scaffold layers were not attached tightly to each other (Figure 1(b)). Moreover, SEM results revealed a greater degree of interlayer deformation in the 100 *μ*m group than in the 200 *μ*m group (Figure 1(c)). Therefore, we chose PCL sheets with 200 *μ*m-thick layers for subsequent *in vitro* cell study analysis. We also used 200 *μ*m-thick layers to fabricate the cage scaffolds (Figure 1(d)). As shown in Figures 1(e) and 1(f), after 0, 1, 3, 5, and 7 days of culture, the extraction fluid showed almost no cytotoxicity (approximately 90% to 95% of cells remained alive in the extract fluid group compared to the control group). The Live/Dead assay revealed a great amount of green-labeled cells (live cells) on the scaffolds, while revealing a scarce amount of red-labeled cells (dead cells) (Figure 1(g)).

### 3.2. Surface Treatment and Properties of the Treated and Untreated Scaffolds

**Figure 2:**
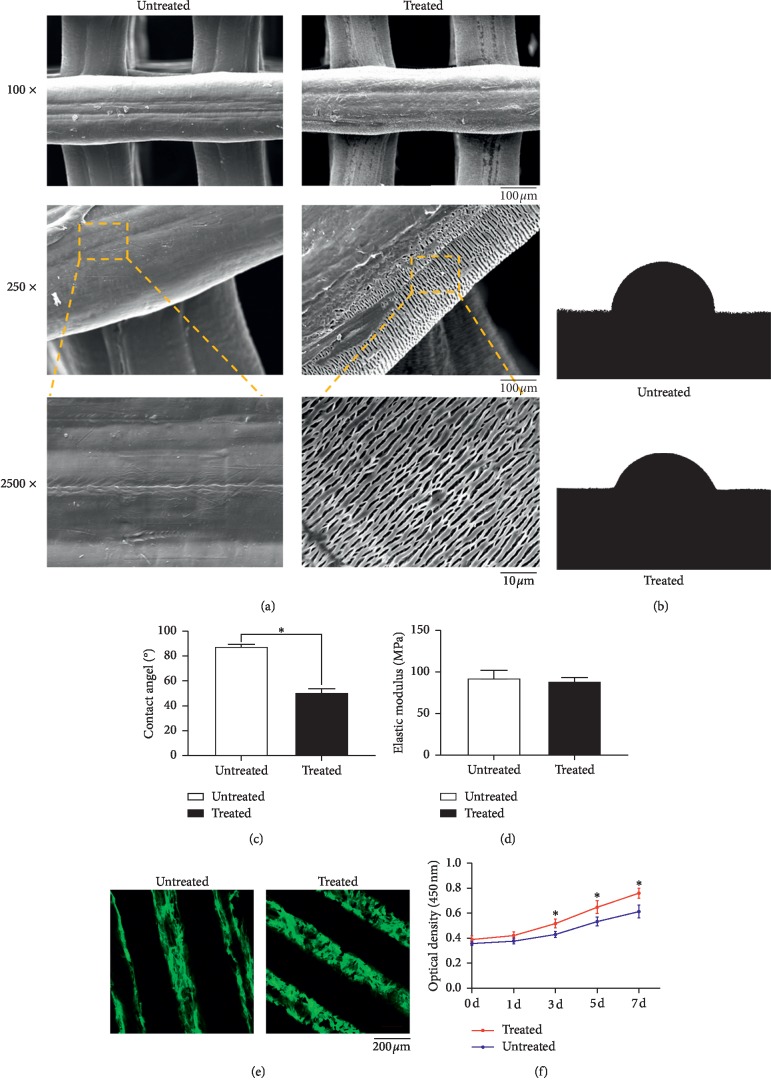
Surface treatment and properties of the treated and untreated scaffolds. (a) Surface roughness of scaffolds treated and untreated with NaOH. Bar represents 100 *μ*m and 10 *μ*m, respectively. (b) Water contact angle images of PCL sheets treated and untreated with NaOH. (c) Summary of the water contact angle. Mean ± SD; *n* = 3; ^*∗*^*p* < 0.05. (d) Elasticity modulus of PCL scaffolds treated and untreated with NaOH. Mean ± SD; *n* = 5. (e) Confocal micrographs of hBMMSCs cultured on treated and untreated PCL sheets for 12 h. Phalloidin is colored green. Bar represents 200 *μ*m. (f) Proliferation curves of hBMMSCs cultured on treated and untreated PCL sheets. Mean ± SD; *n* = 3.

Figure 2(a) shows the PCL scaffold surface, both untreated and treated. Scaffolds in the treated group exhibit a markedly rougher surface than those in the untreated group.

Water-in-air contact angle reflects the hydrophilic/hydrophobic characteristics of a material's surface [12]. Figures 2(b) and 2(c) reveal a contact angle of 87.8° ± 1.40° for untreated PCL scaffolds, and a significant reduction after NaOH treatment (50.5° ± 2.60°). We did not observe any significant differences in elastic moduli between the two groups (Figure 2(d)).

We investigated the adhesion of hBMMSCs on both treated and untreated PCL scaffolds using confocal microscopy (Figure 2(e)). Staining with FITC-phalloidin revealed an increase in hBMMSCs on the treated PCL scaffolds. Cells spread well on both untreated and treated scaffolds and exhibited a fibroblastic morphology with no distinguishable differences. These results suggest that NaOH treatment increases the adhesion ability of hBMMSCs but has little influence on cell morphology. We also observed cell proliferation curves on both untreated and treated PCL scaffolds (Figure 2(f)). These results show significantly increased proliferation in the treated group than the untreated group (*p* < 0.05).

### 3.3. Osteogenic Differentiation of hBMMSCs on PCL Scaffolds

**Figure 3:**
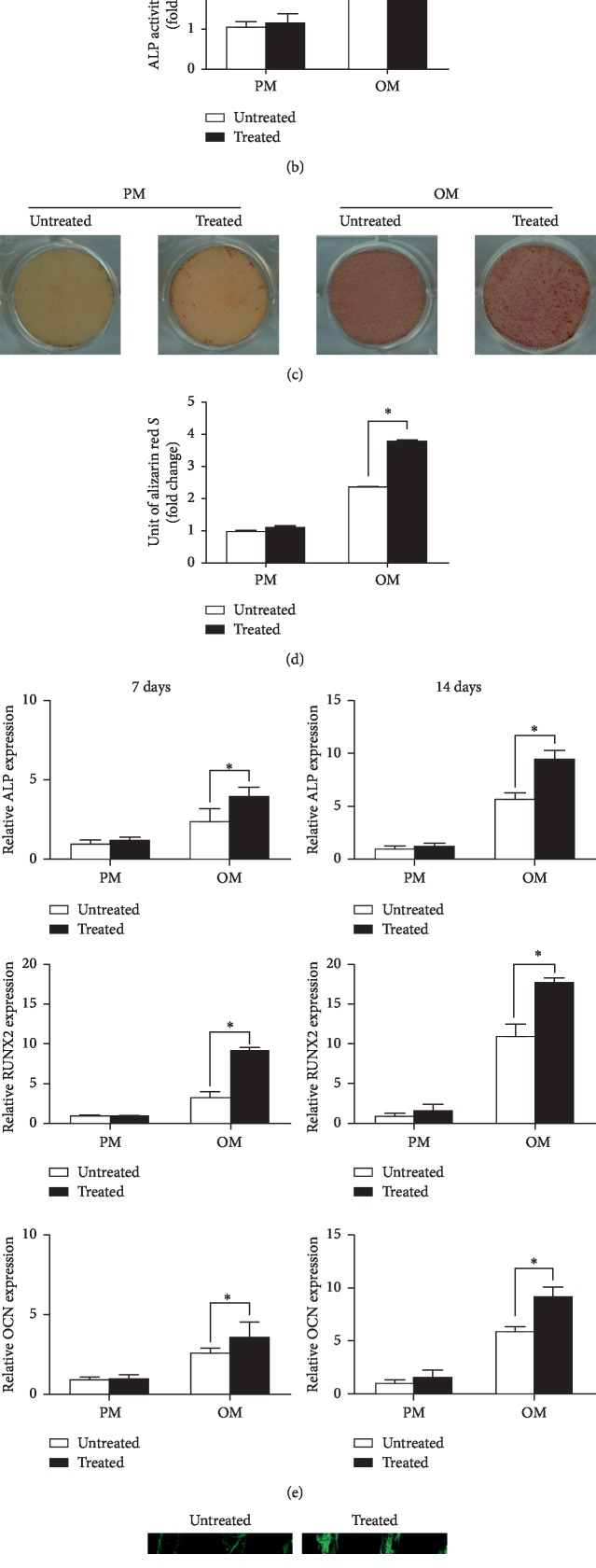
Scaffolds treated with NaOH promoted osteogenic differentiation of hBMMSCs *in vitro*. (a) Alkaline phosphatase (ALP) staining of hBMMSCs cultured on treated and untreated PCL sheets. (b) Quantification of ALP activity of hBMMSCs cultured on treated and untreated PCL sheets 7 days after osteoinduction. Mean ± SD; *n* = 3; ^*∗*^*p* < 0.05. (c) Alizarin red staining of hBMMSCs cultured on treated and untreated PCL sheets. (d) Mineralization assays of hBMMSCs cultured on treated and untreated PCL sheets 14 days after osteoinduction. Mean ± SD; *n* = 3; ^*∗*^*p* < 0.05. (e) Expression of osteogenic genes ALP, RUNX2, and OCN in hBMMSCs cultured for 7 and 14 days after osteoinduction. Mean ± SD; *n* = 3; ^*∗*^*p* < 0.05. (f) Immunofluorescence staining for OCN in hBMMSCs cultured for 14 days after osteoinduction. Bar represents 200 *μ*m.

We tested ALP activity 7 days after OI. Compared to PM, OM raised ALP activity of hBMMSCs on both treated and untreated PCL scaffolds. Furthermore, compared to untreated scaffolds, treatment with NaOH significantly increased ALP activity (*p* < 0.05) in OM (Figures 3(a) and 3(b)). We conducted AR-S staining and quantification of mineralization assays 14 days after OI. We observed minimal calcium levels on scaffolds in PM and more calcium amounts on both types of scaffolds in OM. Moreover, consistent with our ALP activity findings, we observed considerably more calcium on the treated PCL scaffolds (*p* < 0.05) in OM (Figures 3(c) and 3(d)) than on untreated scaffolds.

We measured the gene expression of known osteogenic indices at 7 and 14 days after OI (Figure 3(e)) and showed that hBMMSCs on treated scaffolds had significantly higher osteogenic differentiation in OM. We failed to observe any significant differences in gene expression levels between the two groups in PM.

Moreover, 14 days after OI, immunofluorescent staining showed that cells cultured on treated PCL scaffolds produce more OCN protein (Figure 3(f)).

### 3.4. Ectopic Bone Formation In Vivo

**Figure 4:**
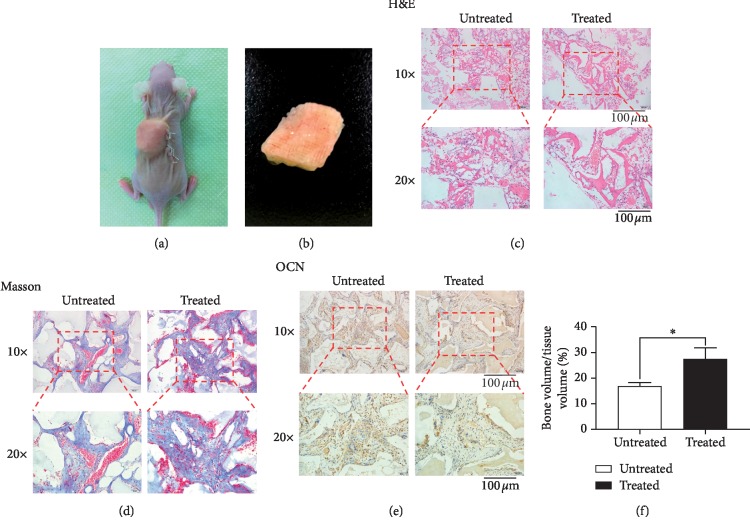
PCL-Bio-Oss hybrid scaffolds treated with NaOH promoted osteogenic differentiation of hBMMSCs *in vivo*. (a) Cage scaffolds were placed into subcutaneous areas of nude mice. (b) Cage scaffolds were harvested at 8 weeks after implantation. (c) H&E staining of treated and untreated groups. (d) Masson's trichrome staining of treated and untreated groups. (e) IHC staining for osteocalcin of treated and untreated groups. (f) Semiquantitative analysis of the staining results. Mean ± SD; *n* = 6; ^*∗*^*p* < 0.05. Bar represents 100 *μ*m for (c), (d), and (e).

We implanted hBMMSC-loaded PCL-Bio-Oss hybrid scaffolds into the dorsal subcutaneous area of nude mice for our *in vivo* analysis (Figure 4(a)). After 8 weeks, we harvested the implants together with their surrounding tissues and analyzed the results. The scaffolds did not show obvious deformation (Figure 4(b)). H&E staining revealed more eosinophilic bone-like tissues in the treated group, which we verified using Masson's trichrome staining (Figures 4(c) and 4(d)). Following IHC staining of OCN, we observed a higher content of dark brown granules in the cytoplasm and around the nuclei in the PCL-NaOH-Bio-Oss group (Figure 4(e)). The result of the histological semiquantitative analysis was consistent with these staining results (Figure 4(f)) and suggests that PCL cage scaffolds loaded with Bio-Oss, especially modified with NaOH, could enhance the osteogenic differentiation of hBMMSCs *in vivo*.

## 4. Discussion

### 4.1. Fabrication of PCL Scaffolds and Parameters Set in the Process

FDM is a popular technique in 3D printing owing to its low cost, processing flexibility, and ease of use [13]. PCL is a filament material commonly used in 3D printing. It is FDA approved and easily processed, and degrades to nontoxic products [14]. However, because of its hydrophobicity, it lacks cell recognition signals and biological adhesion sites, resulting in an unsatisfactory cell response to its surface [15]. To solve this, NaOH surface treatment has been verified as a method to improve the PCL hydrophilicity [16, 17]. It was documented that treatment of NaOH on the PCL surface causes the scission of PCL ester bonds, therefore exposing more carboxyl and hydroxyl groups on the surface, which results in increased hydrophilicity [18, 19]. Consistent with this theory, we reveal that contact angle decreases on the surface of NaOH-treated scaffolds.

Parameters set in the 3D printing process are essential to the properties of scaffolds, such as pore size and layer thickness [20]. We set the pore size at 300 *μ*m as previous studies have shown that the ideal pore size for bone ingrowth is 200–350 *μ*m [21, 22]. With a nozzle size of 300 *μ*m, we found that 200 *μ*m-thick layers formed good connections between filaments with minimal deformation. Furthermore, we acquired hollow scaffolds using an Elements Mixture-I bioprinter.

### 4.2. Effect of NaOH Treatment on Surface Roughness and Mechanical Properties

Our SEM results revealed that PCL scaffolds treated with NaOH exhibited increased surface roughness, and the homogeneous texture turned into meshed structures at the edge of the material filament. We did not observe any notable mechanical degradation in the treated group. Furthermore, the elastic moduli of the scaffolds were within the theoretical human cancellous bone range (50–500 MPa) [23].

### 4.3. Cytotoxicity of PCL and Cell Adhesion, Proliferation on Scaffolds

We chose 75% ethanol to sterilize all the scaffolds in this study. Previous studies have proved that treatment with 75% ethanol for 1 h has no alterations on the morphology and hydrophilicity of PCL scaffolds [24]. We did not observe any obvious cytotoxicity from the PCL material in our confocal microscopy and CCK-8 assay analyses. Evidently, more cells were adhered to the treated scaffolds, which may be due to improved hydrophilicity and surface roughness. In our CCK-8 assay analysis, we observed increased proliferation in the treated group, which may be due to an increase in initial cell adhesion.

### 4.4. Enhanced Osteogenic Differentiation of hBMMSCs on Treated Hybrid Scaffolds

We demonstrated that NaOH-treated PCL scaffolds can promote osteogenic differentiation of hBMMSCs *in vitro*. Namely, we found that expression of osteogenesis-related genes (ALP, RUNX2, and OCN) were upregulated in the treated group in OM medium. 7 days after OI, we noted higher ALP activity in the treated group, which underwent more mineralization after 14 days. OCN protein expression followed the same trend. Previous studies have revealed that surface roughness is a crucial parameter in guiding stem cell osteointegration [25, 26]. Roughness topography may imitate the features of bone surface morphology during bone resorption procedure left by osteoclast activity. The rough surface can also increase the surface area of the scaffolds, allowing greater initial matrix deposition and earlier bone ingrowth [27–29]. Therefore, we hypothesize the increase in surface roughness helps the treated scaffolds promote osteogenic differentiation of hBMMSCs.

Previous groups have produced cage scaffolds using different materials, such as polypropylene and titanium [30–32], which have been fabricated using conventional methods and do not accurately match the parameters applicable to bone tissue engineering. For our *in vivo* experiments, we loaded the cage-shaped scaffolds with Bio-Oss and hBMMSCs and implanted them subcutaneously into nude mice. After 8 weeks, we harvested the scaffolds and found no obvious deformations, indicating the long-term potential of these scaffolds in bone defect repair. We used H&E, Masson's trichrome, and IHC staining to visualize newly formed tissues. These findings showed that NaOH-treated scaffolds enhanced osteogenic differentiation of hBMMSCs *in vivo*. However, this conclusion is restricted to the animal models adopted in our study. Our group is now exploring the role these scaffolds play in osteogenesis, using elaborately designed in situ osteogenesis assessment.

In this study, both pure and NaOH-treated PCL scaffolds demonstrated osteogenesis potential. Previous studies investigating the potential mechanism of osteogenic differentiation on PCL scaffolds have suggested that PCL scaffolds promote osteogenic differentiation of hBMMSCs through both the Wnt/*β*-catenin and Smad3 signaling pathways [8]. Our group plans to verify this in a further study.

## 5. Conclusions

Here, we fabricated hollow cage-shaped PCL scaffolds using the FDM method. We showed that NaOH treatment enhanced hydrophilicity and surface roughness of the scaffolds, which promoted adhesion and osteogenic differentiation of hBMMSCs. Moreover, our PCL-Bio-Oss hybrid scaffold exhibited osteogenic ability *in vivo* with minimal deformation. We manufactured a 3D-printed, surface-modified, PCL-Bio-Oss hybrid scaffold that not only has the potential to promote osteogenesis but can also hold Bio-Oss in a specific site. Therefore, this scaffold has future clinical application potential in repairing specific bone defects.

## Figures and Tables

**Table 1:** Primer pairs used in quantitative real-time PCR.

Gene	Forward primer	Reverse primer
ALP	5′-ATGGGATGGGTGTCTCCACA-3′	5′-CCACGAAGGGGAACTTGTC-3′
RUNX-2	5′-ATGGGATGGGTGTCTCCACA-3′	5′-CCACGAAGGGGAACTTGTC-3′
OCN	5′-CACTCCTCGCCCTATTGGC-3′	5′-CCCTCCTGCTTGGACACAAAG-3′
GAPDH	5′-GAAGGTGAAGGTCGGAGTC-3′	5′-GAAGATGGTGATGGGATTTC-3′

## Data Availability

Data in the present article have been displayed as figures and tables above.
